# TRPV1 Suppresses Microglial Inflammatory Activation to Ameliorate Schizophrenia-Associated Behaviors in Maternal Separation Rats

**DOI:** 10.1093/schbul/sbaf153

**Published:** 2025-09-11

**Authors:** Fashuai Chen, Keke Hao, Chang Shu, Ying Xiong, Rui Xu, Huan Huang, Biwen Peng, Zhongchun Liu, Gavin P Reynolds, Gaohua Wang, Huiling Wang

**Affiliations:** Department of Psychiatry, Central Laboratory, Renmin Hospital of Wuhan University, Wuhan 430060, China; Department of Psychiatry, Central Laboratory, Renmin Hospital of Wuhan University, Wuhan 430060, China; Department of Psychiatry, Nanfang Hospital, Southern Medical University, Guangzhou 510515, China; Department of Psychiatry, Central Laboratory, Renmin Hospital of Wuhan University, Wuhan 430060, China; Department of Psychiatry, Central Laboratory, Renmin Hospital of Wuhan University, Wuhan 430060, China; Department of Psychiatry, Central Laboratory, Renmin Hospital of Wuhan University, Wuhan 430060, China; Department of Psychiatry, Central Laboratory, Renmin Hospital of Wuhan University, Wuhan 430060, China; Department of Physiology, Hubei Provincial Key Laboratory of Developmentally Originated Disorder, School of Basic Medical Sciences, Wuhan University, Wuhan 430071, Hubei, China; Department of Psychiatry, Central Laboratory, Renmin Hospital of Wuhan University, Wuhan 430060, China; Biomolecular Sciences Research Centre, Sheffield Hallam University, Sheffield, United Kingdom; Department of Psychiatry, Central Laboratory, Renmin Hospital of Wuhan University, Wuhan 430060, China; Hubei Provincial Clinical Research Center for Psychiatry, Wuhan 430060, China; Department of Psychiatry, Central Laboratory, Renmin Hospital of Wuhan University, Wuhan 430060, China; Hubei Provincial Clinical Research Center for Psychiatry, Wuhan 430060, China; Hubei Provincial Key Laboratory of Developmentally Originated Disease, Wuhan 430060, China

**Keywords:** Schizophrenia, maternal separation, synaptic plasticity, microglia, TRPV1, CaMKII/NRF2/SIRT3 signaling

## Abstract

**Background and Hypothesis:**

Schizophrenia is linked to hippocampal dysfunction and microglial inflammatory activation. Our prior clinical findings revealed significantly reduced transient receptor potential vanilloid 1 (TRPV1) expression in both first-episode and recurrent schizophrenia patients, with levels inversely correlating with symptom severity, implicating TRPV1 dysfunction in disease progression. Preclinical maternal separation (MS) models recapitulate schizophrenia-like behavioral and synaptic deficits, paralleled by hippocampal microglial TRPV1 downregulation. We hypothesize that early-life stress-induced TRPV1 deficiency in microglia disrupts the calmodulin-dependent protein kinase II (CaMKII)/nuclear factor-erythroid 2-related factor 2 (NRF2)/Sirtuin 3 (SIRT3) signaling axis, thereby amplifying microglial inflammatory responses and synaptic dysfunction underlying cognitive and behavioral impairments.

**Study Design:**

Using a 24-h acute MS model in postnatal day 9 rats, we assessed hippocampal microglial TRPV1 expression, synaptic plasticity, and schizophrenia-like behaviors. Pharmacological (capsaicin, CAP) and genetic (adeno-associated virus (AAV)-mediated overexpression/knockdown (KD)) TRPV1 manipulations were applied. Co-cultures of TRPV1-knockout (KO) microglia and neurons were used to dissect cell-specific effects.

**Study Results:**

MS reduced microglial TRPV1, increased pro-inflammatory cytokines, and induced hyperlocomotion, cognitive deficits, and impaired sensory gating. CAP or microglial TRPV1 overexpression restored synaptic plasticity and reversed behavioral deficits. Conversely, TRPV1 KD worsened neuronal dysfunction. TRPV1-KO microglia, but not neurons, promoted inflammation and neuronal damage via CaMKII/NRF2/SIRT3 downregulation.

**Conclusions:**

These findings provided novel insights into the role of microglial TRPV1 in schizophrenia pathogenesis, establishing it as an upstream regulator of the CaMKII/NRF2/SIRT3 signaling axis—a pathway not previously linked to TRPV1 in neuroinflammation. Our work identifies microglia-specific TRPV1 modulation as a new therapeutic strategy for schizophrenia, highlighting its therapeutic potential for cognitive and negative symptoms in schizophrenia.

## Introduction

Schizophrenia is a severely disabling mental disorder affecting approximately 1% of the global population.^1^^,^^2^ Typically emerging in early adulthood, schizophrenia manifests through positive symptoms, negative symptoms, and cognitive impairments.^3^^,^^4^ While current antipsychotic medications effectively manage positive symptoms, they offer limited benefit for cognitive and negative symptoms.^5^ Cognitive deficits are particularly debilitating and are strongly associated with poor functional outcomes, such as unemployment, impaired social functioning, and reduced independence.^6^^,^^7^ Thus, there is an urgent need to identify novel therapeutic targets that address the cognitive and neurobiological deficits associated with schizophrenia.

Neuroanatomical and neuropathological studies have consistently revealed abnormalities in the hippocampus of individuals with schizophrenia, including reduced hippocampal volume,^8^ neuronal structural damage,^9^ and decreased synaptic density.^10^ These hippocampal abnormalities are strongly linked to impaired learning, memory, and executive functions—core cognitive deficits observed in schizophrenia patients.^11^ Microglia, the resident immune cells of the central nervous system, play an essential role in maintaining neuronal homeostasis through activities such as synaptic pruning, axonal remodeling, and phagocytosis of cellular debris.^12^ Under physiological conditions, homeostatic microglia contribute to healthy neurodevelopment and synaptic function.^13^ However, under pathological conditions, microglia shift toward a disease-associated phenotype, characterized by excessive release of pro-inflammatory cytokines, oxidative stress, and increased phagocytosis of synaptic elements.^14^ Postmortem studies have reported elevated microglial density and increased expression of microglial inflammatory activation markers in the hippocampus and other brain regions of individuals with schizophrenia.^15^^,^^16^ Additionally, positron emission tomography (PET) imaging has demonstrated heightened microglial inflammatory activation in living patients with schizophrenia,^17^ reinforcing the notion that excessive microglial inflammatory activation is a key driver of disease progression. Given the pivotal role of microglial-mediated inflammation in schizophrenia pathogenesis, modulating microglial activity represents a promising therapeutic approach for mitigating cognitive dysfunction and improving clinical outcomes.

Transient receptor potential vanilloid 1 (TRPV1) is a calcium-permeable, nonselective cation channel belonging to the transient receptor potential family.^18^ TRPV1 is widely expressed in sensory neurons and peripheral tissues but has recently been identified in microglia, astrocytes, and other central nervous system cells.^19^^,^^20^ TRPV1 can be activated by various stimuli, including temperature (>43 °C), pH changes, vanilloids (such as capsaicin (CAP)), cannabinoids, and pro-inflammatory lipid mediators.^21^ Our previous clinical study demonstrated that TRPV1 levels were significantly reduced in patients with first-episode schizophrenia and recurrent schizophrenia compared to healthy controls. Moreover, TRPV1 expression was negatively correlated with the severity of schizophrenia symptoms,^22^ which suggests a potential link between TRPV1 dysfunction and schizophrenia pathogenesis.

Emerging evidence suggests that early-life stress (ELS) is a significant environmental risk factor for developing schizophrenia later in life.^23^ Acute maternal separation (MS) in rats is a well-validated model of ELS that recapitulates several schizophrenia-associated behavioral and neuropathological endophenotypes.^14^^,^^24^^,^^25^ Critically, this model demonstrates high face validity, inducing hyperlocomotion, cognitive deficits (working memory, recognition memory, and spatial learning/memory), impaired sensorimotor gating, and hippocampal synaptic dysfunction—core features observed in schizophrenia patients.^4–7^^,^^11^^,^^25^ Furthermore, MS exhibits strong construct validity for schizophrenia pathogenesis: (1) It imposes a neurodevelopmental insult during a vulnerable period, consistent with the neurodevelopmental hypothesis of schizophrenia; (2) It triggers neuroinflammation, specifically microglial inflammatory activation,^14^^,^^24^ which is a well-documented pathological feature in schizophrenia patients via PET imaging and postmortem studies;^15–17^^,^^26^ (3) It induces significant hippocampal pathology (neuronal apoptosis, synaptic loss, plasticity deficits—current results), mirroring hippocampal abnormalities consistently found in schizophrenia;^8–11^ and (4) It replicates our clinical finding of reduced TRPV1 expression,^22^ enhancing its translational relevance. While other models exist, the MS paradigm is particularly relevant as it directly models a developmentally timed environmental stressor known to increase schizophrenia risk in humans, providing a powerful tool to investigate ELS-induced neuroimmune dysregulation and synaptic pathology underlying schizophrenia-associated deficits.

Emerging evidence suggests that TRPV1 plays a critical role in modulating the delicate interactions between microglia and neurons.^27^^,^^28^ Notably, our previous study find that TRPV1 expression is significantly reduced in the hippocampus of MS rats, coinciding with increased neuronal dysfunction and impaired schizophrenia-associated cognitive.^25^ Considering the observed reduction in TRPV1 expression in both schizophrenia patients and MS rats, along with its potential role in microglial function and inflammatory activation, it becomes crucial to determine whether microglial TRPV1 deficiency influences schizophrenia-associated behaviors and to elucidate the underlying molecular mechanisms. Understanding these pathways could provide novel insights into the pathogenesis of schizophrenia and identify potential therapeutic targets for addressing cognitive deficits and microglial inflammatory activation in this disorder.

This study aims to investigate TRPV1 expression in microglia within the hippocampus of MS rats, examine whether pharmacological activation or genetic overexpression of microglial TRPV1 could restore hippocampal synaptic plasticity and improve schizophrenia-associated cognitive and behavioral deficits, and elucidate the underlying molecular mechanisms. Our findings aim to provide novel insights into the role of TRPV1 in schizophrenia pathogenesis and identify potential therapeutic strategies that leverage TRPV1 modulation to ameliorate cognitive impairments and microglial inflammatory activation in this disorder.

## Methods

Detailed descriptions of the materials and methods used in this study are provided in the supplemental information.

## Data Analysis and Statistics

Sample sizes for behavioral, molecular, and histological analyses were determined based on established protocols from our previous studies in this model,^14^^,^^24^^,^^25^ and standard practices in the field, which consistently yield significant effects for the measured parameters. *Post hoc* power analyses confirmed that the chosen sample sizes provided adequate power (typically >0.80) to detect the expected large effect sizes for the primary behavioral and molecular outcomes, based on prior data and pilot studies. The Shapiro–Wilk test and Levene’s test were used to assess the normality of data distribution and homoscedasticity, respectively. The D’Agostino-Pearson test was also employed for normality assessment. Independent samples *t*-tests were used to analyze experiments 1 and 4. Experiments 2 and 3 were analyzed using one-way ANOVA with Bonferroni *post hoc* testing. Repeated-measures data (eg Barnes Maze latency across days) used mixed-effects ANOVA. For data violating normality assumptions, the Kruskal–Wallis test with Dunn’s *post hoc* test was applied. When multiple independent variables were present, two-way ANOVA was performed using Bonferroni *post hoc t*-tests. All data were analyzed by researchers blinded to experimental conditions and are expressed as mean ± SEM. A *P*-value less than .05 (*P* < .05) was considered statistically significant. Statistical significance is denoted as ^*^P < .05, ^**^P < .01, ^***^P < .001, ^****^P < .0001. All statistical analyses were conducted using SPSS software (version 20.0). Specific sample sizes (*n*) for each experiment are provided in the corresponding figure legends.

## Results

### MS-Induced Schizophrenia-Associated Behavior, Hippocampal Neuronal Dysfunction, Microglial Inflammatory Activation, and Microglial TRPV1 Deficiency in Rats

**Figure 1:**
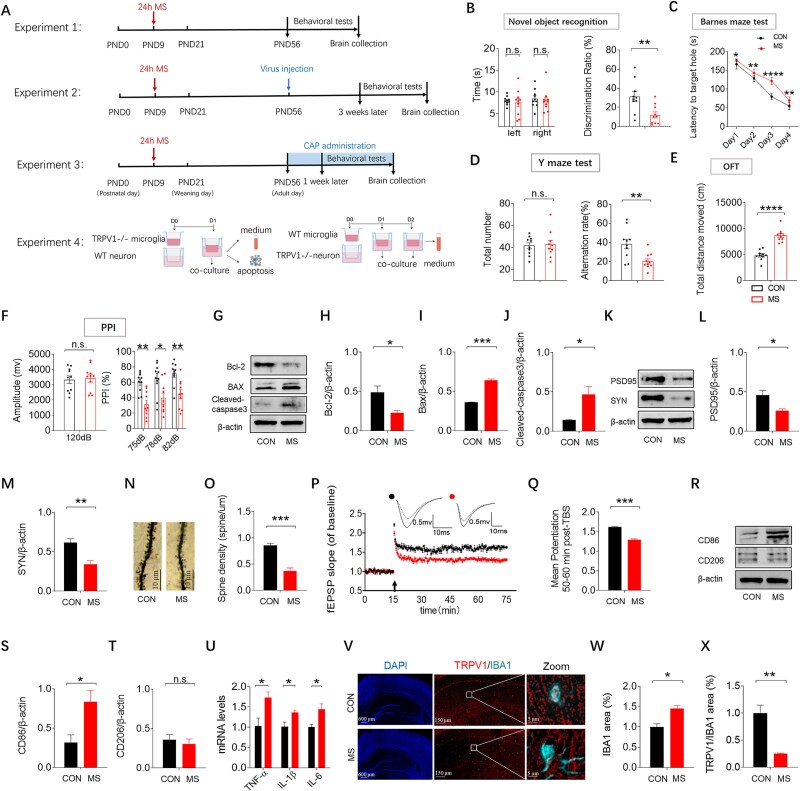
Behavioral Impairments, Increased Apoptosis, Reduced Synaptic Plasticity, Microglial Inflammatory Activation, and Decreased Microglial TRPV1 in MS Rats (A) Experimental workflow for investigating the role of microglial TRPV1 in schizophrenia-associated behaviors. (B) On day 2, rats were allowed 10 min to explore two identical sample objects. On day 3, they spent 10 min exploring a novel and an old object. (C) Latency to enter the target hole in the Barnes maze test. (D) Left: Total number of explorations in the Y-maze test. Right: Spontaneous alternation rate in the Ymaze. (E) Total distance moved in the OFT over 10 min. (F) Left: Baseline startle response to a 120 dB auditory stimulus. Right: Percentage of PPI across various pre-pulse intensities. CON, *n* = 10; MS, *n* = 10 for behavioral testing. (G-J) protein expression of apoptosis-related markers BAX, Bcl-2, and cleaved-caspase3 measured by Western blot in the hippocampus (*n* = 3 per group). (K-M) protein expression of synaptic proteins PSD95 and SYN in the hippocampus measured by Western blot (*n* = 3 per group). (N) Representative images of dendritic spine density in hippocampal CA1 neurons. (O) Quantification of dendritic spine density (*n* = 4 per group). (P) LTP recordings in the hippocampal CA1 region. Arrows indicate time points of TBS application. (Q) Averaged fEPSP slope during the final 10 min of recording (*n* = 4 per group). (R-T) protein expression of microglial inflammatory activation markers CD86 and CD206 in the hippocampus (*n* = 3 per group). (U) The qRT-PCR analysis of pro-inflammatory cytokines TNF-α, IL-1β, and IL-6 in the hippocampus of rats (*n* = 4 per group). (V) Representative immunofluorescence images showing DAPI staining (left), TRPV1 and IBA1 co-localization (middle), and a magnified view of the co-localization (right) in the hippocampal CA1 region. (W) Quantification of IBA1 expression compared to controls (*n* = 4 per group). (X) Quantification of TRPV1 expression compared to controls (*n* = 4 per group). n.s., not significant; TRPV1, transient receptor potential vanilloid 1; MS, maternal separation; fEPSP, field excitatory postsynaptic potential; ^*^P < .05, ^**^P < .01, ^***^P < .001, ^****^P < .0001.

Adult MS rats exhibited significant behavioral impairments. In the NOR test, both MS and control rats spent similar amounts of time exploring two identical objects during the training phase (Figure 1B, left panel). However, 24 h later, MS rats spent significantly less time exploring the novel object compared to controls, indicating deficits in recognition memory (Figure 1B, right panel). In the Barnes maze test, MS rats required more time to locate the target hole across training days 1 to 4, suggesting impaired spatial learning and memory (Figure 1C). Similarly, in the Y-maze test, the spontaneous alternation rate was significantly lower in MS rats, reflecting deficits in working memory (Figure 1D). The OFT showed that MS rats traveled a greater distance than controls, indicating increased locomotor activity (Figure 1E). In the PPI test, baseline startle responses to a 120 dB stimulus were comparable between groups (Figure 1F, left panel). However, MS rats exhibited reduced PPI at various prepulse intensities, suggesting impaired sensorimotor gating (Figure 1F, right panel).

**Table 1:** Specific Primer Sequences for Target Genes

**Gene**	**Sense primer**	**Anti-sense primer**
TNF-α	CTGGCGTGTTCATCCGTTCT	AGCCCATTGGAATCCTTGC
IL-1β	TGTGATGTTCCCATTAGAC	AATACCACTTGTTGGCTTA
IL-6	CCACTGCCTTCCCTACTT	TTGGTCCTTAGCCACTCC
GAPDH	GACATGCCGCCTGGAGAAAC	AGCCCAGGATGCCCTTTAGT

To assess apoptosis in the hippocampus, we examined the expression of key apoptotic markers, including Bcl-2, BAX, and Cleaved-caspase3. Western blot analysis revealed expression of the anti-apoptotic protein Bcl-2 was markedly decreased (Figure 1G and H), and there was a significant increase in the pro-apoptotic proteins BAX and Cleaved-caspase3 in MS rats compared to controls (Figure 1G, I, and  J), which indicates enhanced neuronal apoptosis in MS rats.

We further evaluated the expression of PSD95 and SYN. Western blot results showed significantly reduced PSD95 and SYN levels in the hippocampus of MS rats compared to controls (Figure 1K-M), suggesting hippocampal synaptic dysfunction. Golgi staining demonstrated a significant reduction in dendritic spine density in the hippocampus of MS rats compared to controls (Figure 1N and O). Consistently, electrophysiological recordings revealed impaired long-term potentiation (LTP) at hippocampal CA1 synapses in MS rats (Figure 1P and Q).

To evaluate microglial inflammatory activation in the hippocampus of MS rats, we assessed the expression levels of CD86 and CD206. Western blot analysis revealed a significant increase in CD86 expression in the MS group compared to controls (Figure 1R and S), while CD206 expression remained unchanged between groups (Figure 1R and T). Immunohistochemical staining further showed a marked increase in Iba1-positive microglia in the CA1 region of MS rats, indicative of microglial inflammatory activation (Figure 1V and W). Consistent with these findings, the expression of pro-inflammatory cytokines IL-1β, TNF-α, and IL-6 was significantly elevated in the hippocampus of MS rats (Figure 1U). Notably, microglial TRPV1 expression was significantly reduced in the MS group compared to controls (Figure 1V and X).

### Microglia-Specific TRPV1 Overexpression in the Hippocampal CA1 of MS Rats Ameliorated MS-Induced Microglial Inflammatory Activation, Neuronal Synaptic Dysfunction, and Schizophrenia-Associated Behavioral Impairments

**Figure 2:**
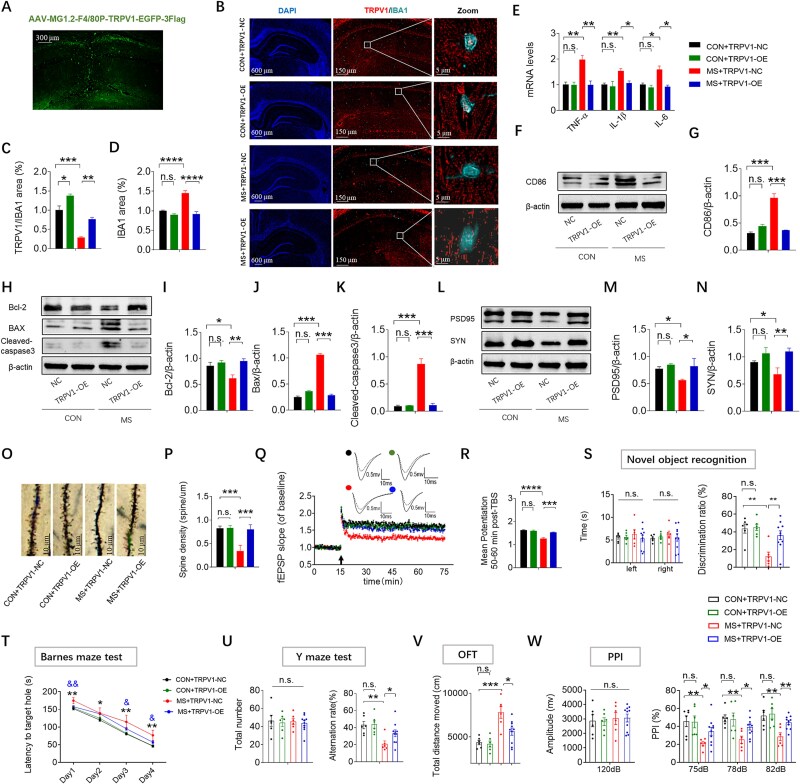
Microglia-Specific TRPV1 Overexpression in the Hippocampal CA1 Ameliorated SchizophreniaAssociated Behaviors in Adult Offspring Exposed to MS (A) Fluorescence images showing efficient expression of AAV-MG1.2-F4/80P-TRPV1-EGFP-3Flag vector in the hippocampal CA1 region. (B) Representative immunofluorescence images showing DAPI staining (left), TRPV1 and IBA1 co-localization (middle), and a magnified view of the co-localization (right) in the hippocampal CA1 region. (C) Quantification of TRPV1 expression in the hippocampus compared to controls (*n* = 4 per group). (D) Quantification of IBA1 expression in the hippocampus compared to controls (*n* = 4 per group). (E) Analysis of TNF-α, IL-1β, and IL-6 levels in the hippocampus (*n* = 4 per group). (F-G) Protein expression of CD86 measured by Western blot in the hippocampus (*n* = 3 per group). (H-K) Protein expression of BAX, Bcl2, and Cleaved-caspase3 measured by Western blot in the hippocampus (*n* = 3 per group). (L-N) Protein expression of PSD95 and SYN measured by Western blot in the hippocampus (*n* = 3 per group). (O) Representative images of dendritic spine density in hippocampal CA1 neurons. (P) Quantification of dendritic spine density (*n* = 6 per group). (Q) LTP recordings in the hippocampal CA1 region. Arrows indicate the time points of TBS application. (R) Averaged fEPSP slope during the last 10 min of LTP recording (*n* = 6 per group). (S) On day 2, time spent exploring two identical sample objects during a 10-min period. On day 3, time spent exploring novel and old objects during a 10-min period. (T) Latency to the target hole in the Barnes maze test. &*P* < .05, &&*P* < .01 as MS+TRPV1-OE rats compared to the MS+TRPV1-NC rats, ^*^P < .05, ^**^P < .01 as CON+TRPV1-NC rats compared to the (U) left: Total number of explorations in the Y-maze. Right: Spontaneous alternation rate in the Y-maze. (V) Total distance moved in the OFT over 10 min. (W) Left: Baseline startle response to a 120 dB auditory stimulus. Right: Percentage of PPI across different pre-pulse intensities. CON+TRPV1-NC, *n* = 6; CON+TRPV1-OE, *n* = 6; MS+TRPV1-NC, *n* = 6; MS+TRPV1-OE, *n* = 10 for behavioral testing. n.s., not significant; TRPV1, transient receptor potential vanilloid 1; MS, maternal separation; TBS, theta burst stimulation; fEPSP, field excitatory postsynaptic potential; ^*^P < .05, ^**^P < .01, ^***^P < .001, ^****^P < .0001.

We overexpressed TRPV1 specifically in microglia using adeno-associated virus (AAV)-MG1.2-F4/80P-TRPV1-EGFP-3Flag injection into the CA1 region, with AAV-MG1.2-F4/80P-NC-EGFP-3Flag serving as a control (Figure 2A). Immunofluorescence analysis confirmed a significant increase in TRPV1 expression in the CON+TRPV1-OE group compared to the CON+TRPV1-NC group, while TRPV1 expression was significantly reduced in the MS+TRPV1-NC group (Figure 2B and C). Importantly, TRPV1 overexpression successfully restored TRPV1 levels in the MS+TRPV1-OE group compared to the MS+TRPV1-NC group (Figure 2B and C).

We next examined microglial activation and inflammatory cytokines across the four groups. Immunofluorescence analysis revealed significantly increased Iba1 fluorescence intensity in the MS+TRPV1-NC group compared to the CON+TRPV1-NC group (Figure 2B and D). However, TRPV1 overexpression in the MS+TRPV1-OE group significantly reduced Iba1 fluorescence intensity compared to the MS+TRPV1-NC group (Figure 2B and D). In addition, tumor necrosis factor-alpha (TNF‑α), interleukin‑1 beta (IL‑1β), and interleukin‑6 (IL‑6) levels were significantly elevated in the MS+TRPV1-NC group compared to the CON+TRPV1-NC group (Figure 2E), but TRPV1 overexpression attenuated the upregulation of these pro-inflammatory cytokines (Figure 2E). Furthermore, TRPV1 overexpression significantly decreased the expression of the M1 microglia marker CD86 in the MS group (Figure 2F and G). These findings suggested that microglial activation and microglial inflammatory activation might be involved in the pathogenesis of early stress-induced schizophrenia mediated by TRPV1.

The expression levels of pro-apoptotic proteins BAX and Cleaved-caspase3 in the hippocampus were significantly elevated in the MS+TRPV1-NC group compared to the CON+TRPV1-NC group (Figure 2H, J, and  K). The expression of the anti-apoptotic protein Bcl-2 was markedly reduced in the MS+TRPV1-NC group compared to the CON+TRPV1-NC group (Figure 2H and I). In the MS+TRPV1-OE group, the expression levels of BAX and Cleaved-caspase3 in the hippocampus were significantly reduced compared to the MS+TRPV1-NC group (Figure 2H, J, and  K). Additionally, the expression of the anti-apoptotic protein Bcl-2 was significantly elevated in the MS+TRPV1-OE group compared to the MS+TRPV1-NC group (Figure 2H and I).

We then measured the concentrations of PSD95 and SYN in the brains of adult rats. Western blot analysis revealed significantly reduced PSD95 and SYN levels in the MS+TRPV1-NC group compared to the CON+TRPV1-NC group (Figure 2L-N). However, TRPV1 overexpression in the MS+TRPV1-OE group markedly increased the expression of both proteins compared to the MS+TRPV1-NC group (Figure 2L-N). Electrophysiological recordings further demonstrated impaired LTP in the MS+TRPV1-NC group (Figure 2Q and R), consistent with synaptic dysfunction. Additionally, Golgi staining revealed significantly reduced dendritic spine density in the MS+TRPV1-NC group compared to the CON+TRPV1-NC group (Figure 2O and P). TRPV1 overexpression mitigated LTP deficits (Figure 2Q and R) and restored dendritic spine density in MS rats (Figure 2O and P), suggesting a protective role of TRPV1 in maintaining synaptic plasticity.

Behavioral assessments indicated that TRPV1 overexpression improved cognitive and behavioral deficits in MS rats. In the OFT, the MS+TRPV1-OE group exhibited reduced total movement distance compared to the MS+TRPV1-NC group (Figure 2V). In the NOR test, the MS+TRPV1-OE group spent more time exploring the novel object compared to the MS+TRPV1-NC group (Figure 2S). In the Barnes maze test, the MS+TRPV1-OE group demonstrated shorter latency to enter the target hole compared to the MS+TRPV1-NC group (Figure 2T). In the Y-maze test, the MS+TRPV1-OE group demonstrated an increased autonomic alternation index compared to the MS+TRPV1-NC group (Figure 2U). In the PPI test, there were no significant differences in baseline responses among the four groups for the 120 dB startle stimulus (Figure 2W, left panel). However, TRPV1 overexpression partially mitigated the impaired PPI responses induced by MS at stimulus levels of 75, 78, and 82 dB (Figure 2W, right panel).

### Microglia-Specific TRPV1 Knockdown in the Hippocampal CA1 of MS Rats Exacerbated MS-Induced Microglial Inflammatory Activation, Neuronal Synaptic Dysfunction, and Schizophrenia-Associated Behavioral impairments

**Figure 3:**
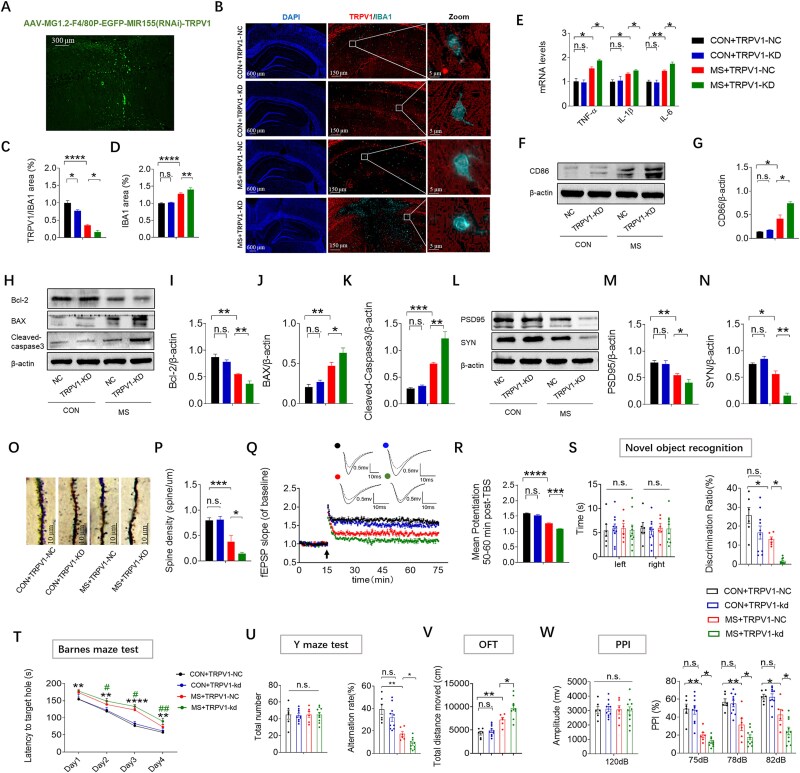
Microglia-Specific TRPV1 KD in the Hippocampal CA1 Exacerbated Schizophrenia-Associated Behaviors in Adult Offspring Exposed to MS (A) Fluorescence images showing efficient expression of AAV-MG1.2-F4/80P-EGFP-MIR155(RNAi)-TRPV1 vector in the hippocampal CA1 region. (B) Representative immunofluorescence images showing DAPI staining (left), TRPV1 and IBA1 co-localization (middle), and a magnified view of the co-localization (right) in the hippocampal CA1 region. (C) Quantification of TRPV1 expression in the hippocampus compared to controls (*n* = 4 per group). (D) Quantification of IBA1 expression in the hippocampus compared to controls (*n* = 4 per group). (E) Analysis of TNF-α, IL-1β, and IL-6 levels in the hippocampus (*n* = 4 per group). (F-G) Protein expression of CD86 measured by Western blot in the hippocampus (*n* = 3 per group). (H-K) protein expression of BAX, Bcl2, and Cleaved-caspase3 measured by Western blot in the hippocampus (*n* = 3 per group). (L-N) Protein expression of PSD95 and SYN measured by Western blot in the hippocampus (*n* = 3 per group). (O) Representative images of dendritic spine density in hippocampal CA1 neurons. (P) Quantification of dendritic spine density (*n* = 6 per group). (Q) LTP recordings in the hippocampal CA1 region. Arrows indicate the time points of TBS application. (R) Averaged fEPSP slope during the last 10 min of LTP recording (*n* = 6 per group). (S) On day 2, time spent exploring two identical sample objects during a 10-min period. On day 3, time spent exploring novel and old objects during a 10-min period. (T) Latency to the target hole in the Barnes maze test. #*P* < .05, ##*P* < .01 as MS+TRPV1-KD rats compared to the MS+TRPV1-NC rats, ^**^P < .01, ^****^P < .0001, as CON+TRPV1-NC rats compared to the MS+TRPV1-NC rats. (U) Left: Total number of explorations in the Y-maze. Right: Spontaneous alternation rate in the Y-maze. (V) Total distance moved in the OFT over 10 min. (W) Left: Baseline startle response to a 120 dB auditory stimulus. Right: Percentage of PPI across different pre-pulse intensities. CON+TRPV1-NC, *n* = 6; CON+TRPV1-KD, *n* = 10; MS+TRPV1-NC, *n* = 6; MS+TRPV1-KD, *n* = 10 for behavioral testing. n.s., not significant; KD, knockdown; TRPV1, transient receptor potential vanilloid 1; MS, maternal separation; TBS, theta burst stimulation; fEPSP, field excitatory postsynaptic potential; ^*^P < .05, ^**^P < .01, ^***^P < .001, ^****^P < .0001.

To investigate the effects of microglial TRPV1 knockdown (KD) in MS rats, we injected AAV-MG1.2-F4/80P-EGFP-MIR155(RNAi)-TRPV1 into the CA1 region to specifically target microglial TRPV1, while AAV-MG1.2-F4/80P-EGFP-MIR155(RNAi)-NC was used as a control (Figure 3A). Immunofluorescence analysis confirmed a significant reduction in TRPV1 expression in both the CON+TRPV1-KD and MS+TRPV1-NC groups compared to the CON+TRPV1-NC group (Figure 3B and C). Furthermore, TRPV1 KD in the MS+TRPV1-KD group led to a further decrease in TRPV1 levels compared to the MS+TRPV1-NC group (Figure 3B and C). Immunofluorescence, Western blotting, and quantitative real-time polymerase chain reaction (qRT‑PCR) were used to assess microglial inflammatory activation across the experimental groups. Iba1 fluorescence intensity was significantly elevated in the MS+TRPV1-NC group compared to the CON+TRPV1-NC group (Figure 3B and D). Notably, TRPV1 KD further amplified Iba1 fluorescence intensity in the MS+TRPV1-KD group relative to the MS+TRPV1-NC group (Figure 3B and D). Additionally, pro-inflammatory cytokine levels (TNF-α, IL-1β, and IL-6) were markedly increased in the MS+TRPV1-NC group compared to the CON+TRPV1-NC group, and TRPV1 KD further elevated these cytokine levels in the MS+TRPV1-KD group (Figure 3E). Similarly, the expression of the M1 microglial marker CD86 was further upregulated in the MS+TRPV1-KD group compared to the MS+TRPV1-NC group (Figure 3F and G).

In the hippocampus, the expression levels of pro-apoptotic proteins BAX and Cleaved-caspase3 were significantly elevated in the MS+TRPV1-NC group compared to the CON+TRPV1-NC group (Figure 3H, J, and K). Conversely, the expression of the anti-apoptotic protein Bcl-2 was markedly reduced in the MS+TRPV1-NC group relative to the CON+TRPV1-NC group (Figure 3H and I). The MS+TRPV1-KD group exhibited increased expression levels of BAX and Cleaved-caspase3 in the hippocampus compared to the MS+TRPV1-NC group (Figure 3H, J, and  K). Furthermore, the expression of the anti-apoptotic protein Bcl-2 was significantly decreased in the MS+TRPV1-KD group compared to the MS+TRPV1-NC group (Figure 3H and I).

We then measured the concentrations of PSD95 and SYN in the brains of adult rats. Western blotting results indicated that the expression levels of hippocampal PSD95 and SYN were significantly reduced in the MS+TRPV1-NC group compared to the CON+TRPV1-NC group (Figure 3L-N). Furthermore, the expression levels of PSD95 and SYN were markedly reduced in the MS+TRPV1-KD group compared to the MS+TRPV1-NC group (Figure 3L-N). Regarding the characteristics of synaptic transmission and plasticity in CA1 synapses, the MS+TRPV1-NC group exhibited impaired LTP (Figure 3Q and R). In line with these findings, neuronal structural analysis revealed a reduced spine density in the MS+TRPV1-NC group compared to the CON+TRPV1-NC group (Figure 3O and P). However, TRPV1 KD further exacerbated the loss of LTP in the MS+TRPV1-KD group relative to the MS+TRPV1-NC group (Figure 3Q and R). Additionally, TRPV1 KD further reduced spine density in the MS+TRPV1-KD group compared to the MS+TRPV1-NC group (Figure 3O and P). Our results suggested that TRPV1 KD might further contribute to the synaptic plasticity dysfunction induced by MS.

Behavioral tests indicated that TRPV1 KD impaired both behavioral and cognitive functions in MS rats. In the OFT, the total distance traveled by the MS+TRPV1-KD group was greater than that of the MS+TRPV1-NC group (Figure 3V). In the NOR test, the MS+TRPV1-KD group spent significantly less time exploring the novel object compared to the MS+TRPV1-NC group (Figure 3S). Additionally, in the Barnes maze test, the MS+TRPV1-KD group exhibited increased latency in entering the target hole compared to the MS+TRPV1-NC group (Figure 3T). In the Y-maze test, the MS+TRPV1-KD group displayed a decreased autonomic alternating circulation index relative to the MS+TRPV1-NC group (Figure 3U). In the PPI test, no significant differences were observed in baseline responses among the four groups for the 120 dB startle stimulus (Figure 3W, left panel). However, TRPV1 KD partially exacerbated the impaired PPI responses induced by MS at 75, 78, and 82 dB (Figure 3W, right panel).

### Knockout of Microglial TRPV1 Promoted Microglial Inflammatory Activation and Induced Co-Cultured Neuronal Injury

**Figure 4:**
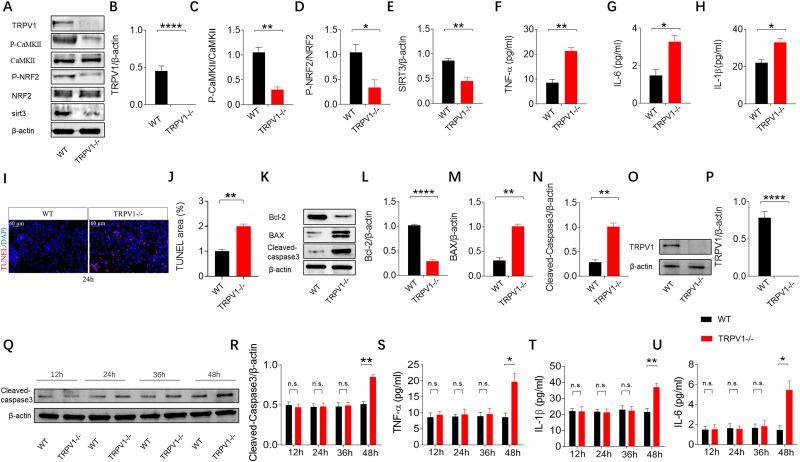
Knockout of Microglial TRPV1 Promoted Microglial Inflammatory Activation and Induced Co-Cultured Neuronal Injury (A-E) Protein expression levels of TRPV1, P-CaMKII, CaMKII, P-NRF2, NRF2, and SIRT3 were measured by Western blot in WT and TRPV1^−/−^ primary microglial cells (*n* = 3 per group). (F-H) secretion of TNF-α, IL-1β, and IL-6 in the supernatants of WT and TRPV1^−/−^ primary microglial cells was measured by enzyme-linked immunosorbent assay (ELISA) (*n* = 4 per group). (I) TUNEL staining showing apoptotic cells (red) in co-cultured primary neuronal cells. DAPI (blue) was used for nuclear labeling (*n* = 4 per group). (J) TUNEL staining (apoptotic cells) and DAPI staining (nuclei) in co-cultured primary neuronal cells (n = 4 per group). (K-N) Protein expression levels of BAX, Bcl2, and Cleaved-caspase3 were measured by Western blot in cocultured primary neuronal cells (*n* = 3 per group). (O-P) protein expression of TRPV1 in WT and TRPV1^−/−^ primary neuronal cells was measured by Western blot (*n* = 3 per group). (Q-R) Protein expression of Cleaved-caspase3 in co-cultured WT primary neuronal cells and TRPV1^−/−^ primary neuronal cells was measured by Western blot (*n* = 3 per group). (S-U) Secretion of TNF-α, IL-1β, and IL-6 in the supernatants of co-cultured WT primary neuronal cells and TRPV1^−/−^ primary neuronal cells at 12, 24, 36, and 48 h was measured by ELISA (*n* = 4 per group). n.s., not significant; TRPV1, transient receptor potential vanilloid 1; p-CaMKII, phosphorylated calmodulin-dependent protein kinase II; CaMKII, calmodulin-dependent protein kinase II; P-NRF2, phosphorylated nuclear factor-erythroid 2-related factor 2; NRF2, factor-erythroid 2-related factor 2; ^*^P < .05, ^**^P < .01, ^***^P < .001, ^****^P < .0001.

Primary microglial cells were isolated from neonatal male WT postnatal day (PND0-PND3) and TRPV1^−/−^ (PND0-PND3) mice on a C57BL/6 background. Western blot analysis showed that TRPV1^−/−^ primary microglial cells exhibited a significant reduction in phosphorylated calmodulin-dependent protein kinase II (p-CaMKII) levels and a decreased p-CaMKII/CaMKII ratio compared to WT microglial cells (Figure 4A and C). Furthermore, we examined the expression of nuclear factor-erythroid 2-related factor 2 (NRF2) and its phosphorylated form (P-NRF2) and found that the P-NRF2/NRF2 ratio was markedly reduced in TRPV1^−/−^ microglia (Figure 4A and D). Additionally, NAD-dependent deacetylase Sirtuin 3 (SIRT3) expression was significantly lower in TRPV1^−/−^ microglial cells compared to WT microglia (Figure 4A and E). Analysis of culture supernatants revealed increased levels of pro-inflammatory cytokines TNF-α, IL-1β, and IL-6 in TRPV1^−/−^ microglia compared to WT microglia (Figure 4F-H). These findings suggested that the TRPV1/CaMKII/NRF2/SIRT3 signaling pathway might regulate microglial inflammatory responses.

To investigate the impact of TRPV1 deficiency on neuron–microglia interactions, primary neuronal cells were co-cultured with either WT or TRPV1^−/−^ microglial cells. TUNEL assay results indicated a significant increase in neuronal apoptosis in the TRPV1^−/−^ microglia co-culture group compared to the WT microglia co-culture group (Figure 4I and J). Moreover, Western blot analysis showed a substantial reduction in the expression of the anti-apoptotic protein Bcl-2 in neurons co-cultured with TRPV1^−/−^ microglia (Figure 4K and L), while the pro-apoptotic proteins BAX and Cleaved-caspase3 were significantly elevated (Figure 4K, M, and  N).

To further assess the reciprocal effects between microglia and neurons, TRPV1^−/−^ and WT neuronal cells were co-cultured separately with WT primary microglial cells. Prior to 48 h of co-culturing, TNF-α, IL-1β, and IL-6 levels in the culture medium did not show significant differences between the two co-culture systems (Figure 4S-U). However, after 48 h of co-culturing, TRPV1^−/−^ neurons exhibited increased Cleaved-caspase3 expression compared to WT neurons (Figure 4Q and R). Additionally, the levels of TNF-α, IL-1β, and IL-6 were significantly elevated in the culture medium of microglia co-cultured with TRPV1^−/−^ neurons compared to those co-cultured with WT neurons (Figure 4S-U).

Collectively, these results indicate that TRPV1 deficiency in microglia exacerbates inflammatory activation and promotes neuronal apoptosis, potentially via the CaMKII/NRF2/SIRT3 signaling pathway. Moreover, TRPV1 deficiency in neurons may further contribute to microglial inflammatory activation, creating a detrimental feedback loop that enhances microglial inflammatory activation responses.

### CAP Administration Inhibited Microglial Inflammatory Activation, Improved Neuronal Synaptic Plasticity, and Schizophrenia-Associated Behaviors in MS Rats

**Figure 5:**
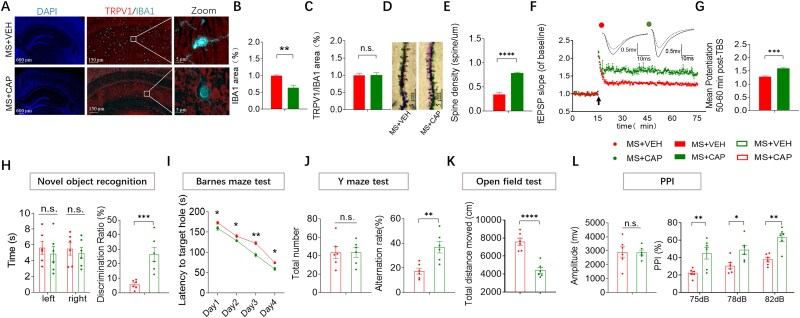
CAP Administration Improved Synaptic Plasticity and Schizophrenia-Associated Behavior in MS Rats (A) Representative immunofluorescence images showing DAPI staining (left), TRPV1 and IBA1 co-localization (middle), and a magnified view of the co-localization (right) in the hippocampal CA1 region. (B) Quantitative analysis of Iba1 levels in the hippocampus compared with the MS+VEH group (*n* = 4 per group). (C) Quantitative analysis of TRPV1 levels in the hippocampus compared with the MS+VEH group (*n* = 4 per group). (D) Representative images of dendritic spine density in hippocampal CA1 neurons. (E) Quantification of dendritic spine density in the hippocampal CA1 region (*n* = 6 per group). (F) LTP recording in the hippocampal CA1 region. Arrows indicate the time point of TBS application. (G) Averaged fEPSP slope during the last 10 min of the recording (*n* = 6 per group). (H) On day 2, the time spent exploring two identical sample objects within a 10-min period. On day 3, the time spent exploring novel and old objects during the 10-min period. (I) Latency to the target hole in the Barnes maze test. (J) Total number of explorations in the Y-maze, shown in the left panel. The Y-maze spontaneous alternation rate is shown in the right panel. (K) Total distance moved in the OFT during a 10-min period. (L) Baseline startle response to an auditory-evoked startle stimulus of 120 dB is shown in the left panel. Percentage PPI of the auditory startle reflex across different pre-pulse intensities is shown in the right panel. MS+VEH (*n* = 10); MS+CAP (*n* = 10) for behavioral testing. n.s., indicates not significant; TRPV1, transient receptor potential vanilloid 1; MS, maternal separation; TBS, theta burst stimulation; fEPSP, field excitatory postsynaptic potential; ^*^P < .05, ^**^P < .01, ^***^P < .001, ^****^P < .0001.

Immunofluorescence analysis revealed a significant reduction in Iba1 fluorescence intensity in the MS+CAP group compared to the MS+VEH group (Figure 5A and B). Notably, CAP administration did not alter TRPV1 levels (Figure 5A and C).

Regarding synaptic transmission and plasticity in CA1 synapses, the MS+VEH group showed impaired LTP (Figure 5F and G). Consistent with these results, neuronal structural analysis revealed a significant reduction in spine density in the MS+VEH group compared to the MS+CAP group (Figure 5D and E). CAP administration significantly mitigated the loss of LTP in the MS+CAP group compared to the MS+VEH group (Figure 5F and G). Additionally, CAP significantly increased spine density in the MS+CAP group compared to the MS+VEH group (Figure 5D and E). These results suggest that CAP administration may protect against synaptic plasticity dysfunction induced by MS.

Behavioral tests demonstrated that CAP administration improved both behavior and cognition in MS rats. In the OFT, the total distance moved by the MS+CAP group was significantly lower than that of the MS+VEH group (Figure 5K). In the NOR test, the MS+CAP group spent more time exploring the new object compared to the MS+VEH group (Figure 5H). In the Barnes maze test, the MS+CAP group showed reduced latency to enter the target hole compared to the MS+VEH group (Figure 5I). In the Y-maze test, the MS+CAP group exhibited a higher autonomic alternating circulation index compared to the MS+VEH group (Figure 5J). In the PPI test, no differences were observed in baseline responses at the 120 dB startle stimulus among the two groups (Figure 5L, left panel). However, CAP administration partially mitigated the impaired PPI responses at 75, 78, and 82 dB in the MS+CAP group compared to the MS+VEH group (Figure 5L, right panel).

## Discussion

The main finding of this study was the identification of microglial TRPV1 as a critical modulator of schizophrenia-associated pathogenesis in an animal model. We observed a significant reduction in microglial TRPV1 expression in the hippocampus, accompanied by decreased neuronal spine density, impaired LTP in the CA1 region, and behavioral abnormalities in MS rats. Notably, overexpression of microglial TRPV1 restored hippocampal synaptic plasticity and ameliorated schizophrenia-associated cognitive and behavioral deficits. Conversely, TRPV1 KD exacerbated neuronal dysfunction and behavioral impairments, highlighting the essential role of microglial TRPV1 in maintaining neurophysiological homeostasis. Administration of CAP, a TRPV1 agonist, mitigated schizophrenia-associated behavioral abnormalities, neuronal apoptosis, and microglial dysfunction in MS rats. Additionally, microglial TRPV1 knockout (KO) resulted in microglia overactivation, disruptions in the CaMKII/NRF2/SIRT3 signaling pathway, and co-culturing neuronal damage, whereas neuronal TRPV1 KO failed to induce microglial inflammatory activation in a co-cultured system. These findings underlined the strong association between schizophrenia-associated behavioral deficits and TRPV1 deficiency-mediated microglial inflammatory activation.

These observations align with our previous findings that SIRT3 downregulation exacerbates microglial activation and cognitive deficits in MS rats.^14^ Here, we identify TRPV1 as the upstream modulator of SIRT3, wherein TRPV1 deficiency disrupts Ca^2+^/CaMKII-dependent NRF2 activation, thereby suppressing SIRT3 expression and its neuroprotective functions. This TRPV1/CaMKII/NRF2/SIRT3 axis represents a unified pathway through which ELS induces neuroinflammation and synaptic impairment.

### Microglial TRPV1 and Inflammatory Activation in Schizophrenia

Microglial cells, as key effectors of the central nervous system’s innate immune response, play an essential role in neurodevelopment and homeostasis.^29^ Previous studies have shown that TRPV1 modulates microglial function under both physiological and pathological conditions. TRPV1 activation has been linked to both pro-inflammatory and anti-inflammatory effects, leading to conflicting reports on its role in neuroinflammation.^30–33^ In this study, we provided strong evidence that TRPV1 deficiency in microglia promoted a pro-inflammatory phenotype, characterized by increased release of inflammatory cytokines. This finding aligns with our previous clinical study, which demonstrated that TRPV1 levels were significantly reduced in patients with first-episode and recurrent schizophrenia compared to healthy controls. Notably, TRPV1 expression was negatively correlated with both schizophrenia symptom severity and inflammatory cytokine levels, suggesting that TRPV1 deficiency may contribute to systemic inflammation in schizophrenia patients.^22^ These findings suggest that TRPV1 plays a crucial role in maintaining microglial homeostasis by suppressing excessive microglial inflammatory activation. This regulatory function may be particularly relevant in schizophrenia, where heightened neuroinflammation is increasingly recognized as a key pathological feature.^26^

Impaired neuronal connectivity and synaptic plasticity are core components of schizophrenia pathogenesis.^34^ Microglial inflammatory activation leads to the release of inflammatory mediators, which can result in neuronal apoptosis and synaptic dysfunction.^35^ Our previous studies have demonstrated increased hippocampal neuronal apoptosis in adult male rats subjected to ELS.^24^^,^^25^ Consistent with these findings, in this study, we observed that co-culturing TRPV1^−/−^ microglia with WT neurons significantly increased inflammatory cytokine levels in the supernatant and exacerbated neuronal apoptosis. However, co-culturing TRPV1^−/−^ neurons with WT microglia did not independently induce microglial inflammatory activation, suggesting that the observed microglial inflammatory activation and neuronal dysfunction are primarily driven by TRPV1 deficiency in microglia rather than neurons.

Inducing a protective microglial phenotype has been proposed as a potential therapeutic strategy for neuropsychiatric disorders.^36^^,^^37^ However, the molecular mechanisms governing microglial functional states remain poorly understood. In this study, we identified TRPV1 as a critical regulator of microglial phenotype in the MS model. Specifically, TRPV1 deficiency led to decreased phosphorylation of CaMKII and NRF2 expression in microglial cells. Additionally, SIRT3 expression was downregulated in TRPV1-deficient microglia. Given that TRPV1 is a calcium-permeable channel known to regulate intracellular Ca^2+^ homeostasis^18^, its deficiency may disrupt calcium signaling and downstream molecular pathways. CaMKII, a key effector of Ca^2+^ signaling, is critically involved in modulating oxidative stress and inflammatory responses.^38^ Furthermore, our previous studies demonstrated that NRF2 and SIRT3 exert protective effects by suppressing microglial activation and enhancing antioxidant defense.^39^^,^^40^ These observations led us to hypothesize that TRPV1 deficiency-induced calcium signaling impairment might underlie the observed changes in CaMKII, NRF2, and SIRT3 expression. Based on this hypothesis, we proposed that TRPV1 may suppress microglial inflammatory activation via the Ca^2+^/CaMKII/NRF2/SIRT3 signaling pathway, offering a novel mechanism for schizophrenia pathogenesis and potential therapeutic strategies.

### TRPV1 as a Therapeutic Target for Schizophrenia

Given its crucial role in modulating microglial inflammatory activation and synaptic plasticity, TRPV1 emerges as a promising therapeutic target for schizophrenia. Importantly, our previous clinical study demonstrated reduced TRPV1 expression in schizophrenia patients,^22^ supporting its potential as both a therapeutic target and a peripheral biomarker for disease severity. Conventional antipsychotics primarily alleviate positive symptoms, while cognitive impairments remain largely unaddressed.^41^ Recent advances in immunomodulatory therapies, including those targeting microglial inflammatory activation, have shown promise in treating neuropsychiatric disorders.^36^ Our findings highlight microglial TRPV1 activation as a novel approach to simultaneously regulate microglial inflammatory activation and restore synaptic plasticity, offering a dual-benefit therapeutic strategy for schizophrenia.

Notably, CAP, a TRPV1 agonist, successfully mitigated schizophrenia-associated deficits in our study, suggesting the potential of pharmacological TRPV1 activation as a novel intervention. However, systemic TRPV1 activation could lead to adverse effects, such as pain hypersensitivity and thermoregulation disturbances.^42^ Therefore, developing microglial-specific TRPV1 modulators or targeted gene therapies could provide more selective approaches with reduced off-target effects. Further studies are necessary to explore the long-term effects of TRPV1 modulation in microglia, as well as the feasibility of developing selective TRPV1 agonists or gene therapies that can specifically enhance microglial TRPV1 expression. These studies will help elucidate the therapeutic potential of microglial TRPV1 in treating schizophrenia and other neuroinflammatory disorders.

Our findings support two potential clinical applications: (1) Preventive Strategy: ELS is a recognized risk factor for schizophrenia. The observed downregulation of microglial TRPV1 in the MS model—occurring alongside neurodevelopmental insults—suggests that interventions during prodromal or high-risk stages (eg in individuals with ELS histories) could mitigate microglial priming and subsequent neuroinflammation. TRPV1 activation during vulnerable periods (eg adolescence) might prevent the emergence of hippocampal pathology and cognitive deficits. (2) Therapeutic Strategy: Pharmacological TRPV1 activation (eg CAP) restored synaptic plasticity and ameliorated behavioral deficits in adult MS rats with established pathology. This supports targeting TRPV1 in symptomatic patients, particularly those with prominent cognitive deficits and neuroinflammation. TRPV1 expression (as a peripheral biomarker^22^) could help identify patients most likely to benefit. While both approaches show promise, key questions remain: Would TRPV1 modulation in early prodromal stages prevent transition to psychosis? Can it reverse chronic synaptic deficits in late-stage schizophrenia? Future studies should evaluate TRPV1 interventions across disease stages and explore combinatorial approaches with immunomodulatory agents.

Despite strong evidence supporting microglial TRPV1 as a key regulator of schizophrenia pathogenesis, several limitations must be acknowledged. First, our study focused exclusively on male rats to avoid potential confounding effects of estrogen on neuronal activity, microglial function, and behavioral outcomes.^43^^,^^44^ Given that sex differences exist in microglial function,^45^^,^^46^ future research should investigate TRPV1 regulation and its therapeutic potential in female models. Behavioral tests were conducted sequentially rather than counterbalanced. Although identical conditions and rest intervals minimize bias, future studies could employ randomized test sequences to exclude carryover effects. Additionally, while our findings suggest that TRPV1 activation mitigates schizophrenia-associated pathogenesis, further studies are required to evaluate its long-term effects and potential interactions with existing antipsychotic treatments.

## Conclusions

**Figure 6:**
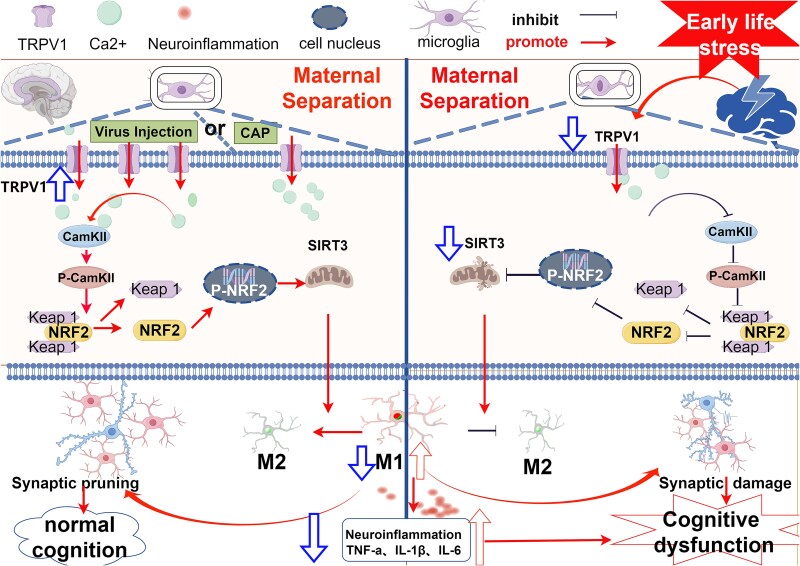
The Proposed Mechanism of TRPV1 in Cognitive Impairment in MS Rats. In the MS model, microglial TRPV1 deficiency leads to downregulation of the CaMKII/NRF2/SIRT3 signaling pathway. This downregulation results in enhanced microglial inflammatory activation (M1), characterized by increased release of pro-inflammatory cytokines. These pathological changes contribute to synaptic loss, impaired hippocampal plasticity, and ultimately, cognitive deficits. Conversely, pharmacological activation or overexpression of TRPV1 restores CaMKII/NRF2/SIRT3 signaling, suppresses microglial inflammatory activation (M2), rescues hippocampal synaptic plasticity, improving cognitive and behavioral outcomes. This mechanistic pathway highlights TRPV1 as a potential therapeutic target for mitigating schizophrenia-associated cognitive impairments in MS rats. MS, maternal separation; TRPV1, transient receptor potential vanilloid 1; CaMKII, calmodulin-dependent protein kinase II; NRF2, factor-erythroid 2-related factor 2.

In summary, our study provides novel insights into the role of TRPV1 in microglial inflammatory activation in schizophrenia. We demonstrate that microglial TRPV1 expression is reduced in MS rats, and its activation via pharmacological or genetic means ameliorates schizophrenia-associated behavioral deficits and synaptic plasticity impairments as shown in Figure 6. Furthermore, our findings highlight TRPV1 as a potential therapeutic target, offering a new avenue for developing microglia-focused treatments for schizophrenia. Future research should focus on developing microglia-specific TRPV1 modulators and further elucidating the long-term therapeutic benefits and safety of TRPV1-based interventions.

## Supplementary Material

Revised_Supplemental_information_sbaf153

## Data Availability

Data generated during the current study are available from the corresponding author upon reasonable request.
