# Supplementary material for: TRPV1 Suppresses Microglial Inflammatory Activation to Ameliorate Schizophrenia-Associated Behaviors in Maternal Separation Rats
Source: Schizophr Bull. 2025 Sep 11;52(4):sbaf153. doi: 10.1093/schbul/sbaf153 (PMC13391685; doi:10.1093/schbul/sbaf153)
Supplement: Revised_Supplemental_information_sbaf153 [file revised_supplemental_information_sbaf153.docx]

**Materials and methods**

1. ***Animals and MS***

Forty nulliparous female and forty male eight-week-old Wistar rats were obtained from Beijing Vital Rival Laboratory Animal Technology Co., Ltd. (Beijing, China). Rats of the same sex were housed in groups of 3 or 4 per cage. At 3 months of age, the animals were mated, with the males removed one week later. Pregnant females were housed individually in ventilated plastic cages in a temperature- and humidity-controlled facility (25 ± 2°C, 50 ± 10% humidity), with a constant 12-hour light–dark cycle (lights on: 08:00–20:00). All animals had free access to food and tap water. The MS protocols were performed according to previous research [1]. Females were checked twice daily for delivery (08:00 and 17:00), with the day of delivery defined as post-natal day (PND) 0. Each pregnant rat gave birth to an average of 10 ± 2 pups. On PND 9, litters were randomly assigned to either the MS or control groups. In the MS group, mothers were removed at 10:00 AM, and the pups were left in their home cages with a heated mat for 24 hours before the mothers were returned. The control group pups grew naturally with their mothers. All litters were otherwise left undisturbed, except for routine cage cleaning. On PND 21, the MS and control pups were weaned and group-housed by sex (3–4 per cage). Since previous studies have shown that estrogen plays a key role in regulating neuronal activity and animal behavior and alterations in estrogen signaling are linked to a range of neurological and psychiatric conditions [2, 3], all subsequent experiments were carried out only on male offspring. All procedures involving animals were approved by the Institutional Animal Care and Use Committee of Renmin Hospital of Wuhan University.

B6.129X1-TRPV1 knockout (TRPV1^-/-^) mice were purchased from Jackson Laboratory, and wild-type (WT) mice were provided by the Hubei Province Center for Animal Experiments. The mice were housed in groups and maintained on a 12-hour light–dark cycle in a room at 25 ± 2°C with a relative humidity of 60–80%. Both the KO and WT mice were on a C57BL/6J background. Animal experiments were conducted according to protocols approved by the Care and Use Committee of Wuhan University Medical School. All mice were housed in the Animal Biosafety Level III Laboratory (ABSL-III) of Wuhan University.

1. ***Surgery***

Rats were deeply anesthetized with 3% sodium pentobarbital (30 mg/kg body weight) and fixed in a stereotactic frame. The virus was injected into the hippocampal CA1 region (1 μl) [AP, −3.96 mm from bregma; ML, ±2.40 mm; DV, −2.80 mm from the dura for AAV-MG1.2-F4/80P-TRPV1-EGFP-3Flag and AAV-MG1.2-F4/80P-EGFP-MIR155(RNAi)-TRPV1 viruses] using a 10 μl glass syringe with a fixed needle at an injection rate of 0.1 μl/min. The injection needle was left in place for an additional 10 minutes following the injection to allow for proper diffusion of the virus. After surgery, rats were allowed to recover from anesthesia on a heating pad.

1. ***Experimental design***

Experiment 1: On PND 9, dams and their pups were randomly assigned to either the control group or the MS group. Behavioral and molecular biology tests were performed when rats reached adulthood. The experimental design is depicted in Fig. 1A.

Experiment 2: AAV-MG1.2-F4/80P-TRPV1-EGFP-3Flag and AAV-MG1.2-F4/80P-EGFP-MIR155(RNAi)-TRPV1 viruses were stereotactically injected into rats at PND56 under 3% sodium pentobarbital anesthesia. Viral vectors were injected bilaterally into the hippocampal CA1 region (coordinates: AP −3.96 mm, ML ±2.40 mm, DV −2.80 mm from bregma). Three weeks following the stereotactic injections, behavioral and molecular biology tests were conducted on the rats. The experimental design is shown in Fig. 1A.

Experiment 3: On PND 9, dams and their pups were randomly assigned to either the control group or the MS group. For the MS+VEH group, rats received a vehicle treatment (1 ml/kg/day, intraperitoneal injection), which consisted of a 1:1:8 mixture of Tween 80: ethanol: saline. Rats in the MS+CAP group were treated with CAP (1 mg/kg, intraperitoneal injection; single injection per day) for 1 week prior to behavioral testing, and the treatment continued during the behavioral tests. This dose was selected based on: (1) our prior study demonstrating its efficacy in rescuing neuronal apoptosis and schizophrenia-like behavioral deficits in a maternal separation (MS) model without adverse effects [4]; (2) pharmacokinetic and receptor-binding studies confirming that this dose achieves brain concentrations sufficient to activate TRPV1 while avoiding nociceptive side effects [5, 6]; (3) established protocols for TRPV1-targeted neuroprotection in rodent models of neuropsychiatric disorders [7, 8]. CAP was purchased from Sigma-Aldrich and dissolved in the 1:1:8 mixture of Tween 80: ethanol: saline. The experimental design is illustrated in Fig. 1A.

Experiment 4: Primary hippocampal microglial and neuronal cells were isolated from neonatal male WT mice (PND0–PND3) and TRPV1^-/-^ mice (PND0–PND3) with a C57BL/6 background. TRPV1^-/-^ primary microglial cells were co-cultured with WT primary neuronal cells, while TRPV1^-/-^ primary neuronal cells were co-cultured with WT primary microglial cells. The expression of signaling pathway proteins, levels of inflammatory cytokines, and cell apoptosis were assessed separately. The experimental design is shown in Fig. 1A.

1. ***Behavioral testing of animals***

Behavioral evaluations were conducted during the adult stage of development. The number of animals used for testing was determined based on prior publications and expertise in phenotyping. Prior to all tests, animals were allowed a 30-minute acclimation period in the behavioral room. Behavioral assessments were conducted during the light phase (08:00–18:00) to standardize testing conditions relative to the rodents' circadian rhythm. This timing minimizes confounding effects of natural nocturnal hyperactivity, enhances detection sensitivity for behavioral deficits, and reduces stress associated with handling during the resting period. Various behavioral measures were assessed for each rat. Following each trial, the apparatus was cleaned with 75% alcohol.

- 1. ***Open-field Test (OFT)***

The OFT is widely used to evaluate spontaneous locomotor activity in rodents [9]. In our study, the OFT was used to measure spontaneous locomotor activity in rats. Detailed protocols for this test have been provided in our previously published papers [10, 11].

- 1. ***Novel object recognition (NOR) test***

The NOR test is used to assess changes in recognition memory of the experimental animals [12]. In this study, the NOR test was utilized to assess changes in recognition memory in rats. Throughout all phases of the NOR test (habituation, training, and testing), rats had free access to food and water in their home cages. More detailed information on the test procedure can be found in our previously published papers [10, 11].

- 1. ***Barnes maze test***

The Barnes maze test is widely used to evaluate hippocampus-dependent spatial memory in rodents [13]. We employed this test to examine spatial memory in rats. For further details on the test procedure, refer to our previously published papers [10, 11].

- 1. ***Y-mase test***

The Y-maze test is used to evaluate attention and short-term working memory of rodents [14]. The Y-maze apparatus consists of three black arms arranged at 120-degree angles, each with a length of 50 cm, width of 10 cm, and height of 30 cm. The arms converge at a central region, and the structure is mounted 50 cm above the ground. After placing the rats in the apparatus, their movements were recorded by video and analyzed using EthoVision software. In the autonomous alternation test, each rat was placed at the starting arm and allowed to explore for 8 minutes. Alternation rate %= number of alternations / (total number -2) %.

- 1. ***Pre-pulse inhibition (PPI) test***

The PPI test is a well-established method to evaluate sensorimotor gating deficits, a core symptom of schizophrenia, in rodents [15]. We used the PPI test to assess schizophrenia-related behaviors in rats. Detailed methodology for this test is provided in our previously published papers [10, 11].

1. ***Sample collection***

Following behavioral testing, animals were humanely euthanized under anesthesia with 1% pentobarbital sodium. Subsequently, rat hippocampal tissues were collected from each group, snap-frozen over dry ice, and stored at -80°C until required for dissection and analysis of gene and protein expression.

1. ***Protein extraction and western blot analysis***

Tissue dissected without perfusion. Total protein was extracted from the hippocampal tissues using RIPA buffer (P0013B, Beyotime Biotech) on ice, supplemented with 100 mM phenylmethanesulfonyl fluoride (ST506, Beyotime Biotech) and Protease/Phosphatase Inhibitor (Servicebio, Wuhan). The protein concentration was determined using a BCA kit (P0010S, Beyotime Biotech). Equal amounts of RIPA-extracted protein were loaded and separated using an 8% SDS-PAGE gel, then transferred to polyvinylidene fluoride (PVDF) membranes. Western blotting was conducted using the following primary antibodies: Mouse anti-TRPV1 (dilution 1:800, ab203103, Abcam, Cambridge, UK), rabbit anti-Phospho- Calcium/calmodulin-dependent protein kinase II (CaMKII) (dilution 1:1000,#12716S, Cell Signaling Technology), rabbit anti-CaMKII (dilution 1:2000, #12666-2-AP, Proteintech) , rabbit anti- Nuclear factor erythroid 2-related factor 2 (NRF2) (dilution 1:1000, ab62352, Abcam, Cambridge, UK), rabbit anti-Phospho-NRF2 (dilution 1:1000, DF7519, Affinity Biosciences, China), rabbit anti-CD86 (dilution 1:1000, #13395-1-AP, Proteintech), rabbit CD206 (dilution 1:500, #18704-1- AP, Proteintech), rabbit anti- sirtuin-3 (SIRT3) (dilution 1:1000, #10099-1-AP, Proteintech), rabbit anti-BAX (dilution 1:1000, GB11690, Servicebio, China), rabbit anti-Bcl2 (dilution 1:1000, abs147821, Absin, China), rabbit anti-Cleaved-caspase3 (dilution 1:2000, abs132005, Absin, China), rabbit anti-PSD95 (dilution 1:1000, ab238135, Abcam, Cambridge, UK), rabbit anti- Synaptophysin (dilution 1:1000, ab52636, Abcam, Cambridge, UK), and rabbit anti-β-actin (dilution 1:1000, ab181602, Abcam, UK). The following secondary horseradish peroxidase (HRP)-conjugated antibodies were used at 1:5000 dilution: goat anti-rabbit HRP (12–348, Millipore, USA) and goat anti-mouse HRP (sc-2005, Santa Cruz Biotechnology, USA). Protein bands were visualized using enhanced chemiluminescence detection reagents and captured with a Chemidoc™ Touch Imaging System (Bio-Rad, Hercules, CA, USA).

1. ***RNA Preparation and Gene Expression Analysis by Quantitative Real-Time Polymerase Chain Reaction (qRT-PCR)***

Tissue dissected without perfusion. Total RNA was extracted from hippocampal tissues using TRIzol reagent (Invitrogen, Carlsbad, CA, USA) according to the manufacturer’s instructions. RNA was further purified by chloroform extraction and isopropanol precipitation. The concentration and purity of RNA were determined using a NanoDrop 2000 spectrophotometer (Thermo Fisher Scientific, Waltham, MA, USA). Then, 5 μg of total RNA was reverse transcribed into cDNA using a PrimeScript RT Kit (RR820A, Takara Bio Inc., Shiga, Japan). The reverse transcription reaction was performed at 42°C for 1 hour, heated to 70°C for 5 minutes, and then cooled to 48°C. For qRT-PCR, a 20 μl reaction mixture was prepared with SYBR Master Mix, and 1 μl each of forward and reverse primers was added. qRT-PCR conditions were: 95°C for 30 seconds, followed by 40 cycles of 95°C for 5 seconds and 60°C for 30 seconds, then 95°C for 10 seconds and 60°C for 5 seconds. The 2^−ΔΔCT^ method was used to calculate mRNA expression levels, with GAPDH as the reference gene. Primer sequences used in the qRT-PCR assay are listed in Table 1.

1. ***Immunofluorescent staining assay***

Following anesthesia, rats were perfused through the heart with PBS, followed by 4% paraformaldehyde. Brains were fixed overnight in 4% paraformaldehyde, embedded in paraffin, and sectioned at 40 μm thickness. The paraffin-embedded tissue sections were dewaxed, rehydrated, and subjected to antigen retrieval using sodium citrate and microwaving. Sections were then blocked with 1% BSA (Roche, Basel, Switzerland) for 2 hours at room temperature. The sections were incubated overnight at 4°C with primary antibodies: mouse anti-TRPV1 (dilution 1:5000, ab203103, Abcam, Cambridge, UK) and rabbit anti-IBA1 (dilution 1:5000, GB113502, Servicebio, China). The corresponding HRP-conjugated secondary antibodies and Tyramide Signal Amplification (TSA) dye (iF555-Tyramide, dilution 1:500, G1233, Servicebio, China; iF440-Tyramide, dilution 1:500, G1250, Servicebio, China) were applied. Nuclei were counterstained with DAPI (Thermo Fisher Scientific, Waltham, USA). Images were captured using a fluorescence microscope (IX53, Olympus, Tokyo, Japan).

1. ***Golgi-cox staining***

At the conclusion of the behavioral tests, four rats were randomly selected from each group. The brains were swiftly extracted from deeply anesthetized rats. Brains extracted without perfusion. Golgi-Cox staining was performed using the FD Rapid Golgi Stain™ Kit (FD Neuro Technologies Inc., USA) according to the manufacturer's guidelines. Coronal brain sections, 150 μm thick, were obtained using a Leica VT1200S vibratome (Germany) and mounted on gelatin-coated slides. Following the kit's protocol, the sections were processed and stained for analysis of dendritic spine morphology. Microscopic images were captured using a fluorescence microscope, and the images were analyzed using NIH ImageJ software.

1. ***Electrophysiology***

Following anesthesia with 1% pentobarbital sodium, the brains of rats were rapidly extracted and sliced in chilled artificial cerebrospinal fluid (ACSF) with the following composition (in mM): NaCl 125, KCl 3, NaH2PO4 1.25, NaHCO3 25, CaCl2 2, MgSO4 1, glucose 10. Fresh brain slices prepared without perfusion. The ACSF was pre-saturated with 95% O2 and 5% CO2. Horizontal slices (340 μm thick) were prepared using a Leica VT1000S vibratome. These slices were incubated at 32°C for 30 minutes in ACSF and then transferred to a recording chamber perfused continuously with externally filtered ACSF at a flow rate of 2 ml/min at room temperature. Field excitatory postsynaptic potentials (fEPSPs) were recorded from CA1 stratum radiatum apical dendrites using a glass pipette filled with 2 M NaCl (1–2 MΩ), in response to orthodromic stimulation of Schaffer collateral-commissural projections in CA1. Stimuli were delivered at a rate of 0.05 Hz, with a pulse current that elicited a 50% maximal spike-free response. After a stable baseline (15 minutes) was established, long-term potentiation (LTP) was induced by theta burst stimulation (TBS), consisting of five bursts, each with four pulses at 100 Hz separated by 200 ms. Data were acquired and digitized using the NAC 2.0 Neurodata Acquisition System (Theta Burst Corp., Irvine, CA), and stored for analysis. Data are presented as means ± SEM, with the average fEPSP slope during the last 10 minutes of the recording used for analysis.

1. ***Primary cells culture***

Primary microglial cells were isolated from WT mice (PND0–PND3) and TRPV1^-/-^ mice (PND0–PND3) with a C57BL/6 background, as previously described [16]. In brief, the brains from newborn mice were washed in 75% alcohol, and the whole brains were isolated and minced in pre-cooled PBS. The cortical tissue was then digested for 20 minutes using 0.25% trypsin, followed by centrifugation and resuspension. The samples were digested with DNase I at 37°C and subsequently transferred to a single-cell suspension. The cells were plated onto poly-D-lysine-coated flasks and cultured for 14 days. Microglial cells were isolated from mixed glial cultures by shaking the flasks at 200 rpm for 3 hours. The experimental flowchart is depicted in Fig. 1A.

Primary neuronal cells were prepared as described previously [17]. Briefly, hippocampal tissue was isolated from WT and TRPV1^-/-^ C57BL/6 mice on PND0. Dissociated cells were maintained in DMEM supplemented with 10% fetal bovine serum (FBS, HyClone, Logan, UT, USA) and plated onto poly-D-lysine-coated 6-well plates at a density of 1 × 10^6 cells per well. After 4 hours of incubation, the medium was replaced with neurobasal medium (Gibco) supplemented with 2% B27 (Gibco). Neuronal cultures were maintained in a humidified atmosphere at 37°C with 5% CO2. Neurons from days 5 to 7 of in vitro culture were used for experiments. The experimental flowchart is shown in Fig. 1A.

***11.1 ELISA assay***

Inflammatory cytokine levels (IL-1β, IL-6, TNF-α) in the supernatants of primary microglial cell cultures were quantified using enzyme-linked immunosorbent assay (ELISA) kits (Neobioscience Technology, Shanghai, China) following the manufacturer's protocol. Absorbance was measured at 450 nm using a Microplate reader (Spectra Max i3x, Molecular Devices, USA) to assess cytokine production.

***11.2 TUNEL assay***

Cell cultures were fixed in 4% paraformaldehyde for 30 minutes and then incubated with a TUNEL reaction mixture for 1 to 1.5 hours. Sections were developed using peroxidase solution and diaminobenzidine, followed by counterstaining with hematoxylin. The sections were dehydrated in gradient alcohol, permeabilized, and sealed with neutral gum. DAPI (Thermo Fisher Scientific) was used for nuclear staining. Images were captured using an inverted fluorescence microscope (Olympus BX51, Olympus, Tokyo, Japan), and ImageJ (National Institutes of Health, Bethesda, MD) was used to analyze the integral optical density (IOD) of the target protein.

***11.3 Protein extraction and western blot analysis***

Total protein was extracted from cultured primary neuronal and microglial cells using a protein lysis buffer (Beyotime Biotech, China). Protein concentration was determined using a BCA kit (P0010S, Beyotime Biotech). The proteins were separated by 10% SDS-polyacrylamide gel electrophoresis and transferred onto PVDF membranes. Western blot analysis was performed using the following primary antibodies: Mouse anti-TRPV1 (1:800, ab203103, Abcam, Cambridge, UK), rabbit anti-Phospho-CaMKII (1:1000, #12716S, Cell Signaling Technology), rabbit anti-CaMKII (1:2000, #12666-2-AP, Proteintech), rabbit anti-Phospho-NRF2 (1:1000, DF7519, Affinity Biosciences, China), rabbit anti-NRF2 (1:1000, ab62352, Abcam, Cambridge, UK), rabbit anti-Sirt3 (1:1000, #10099-1-AP, Proteintech), rabbit anti-BAX (1:1000, GB11690, Servicebio, China), rabbit anti-Bcl2 (1:1000, abs147821, Absin, China), rabbit anti-Cleaved-caspase3 (1:2000, abs132005, Absin, China), and rabbit anti-β-actin (1:1000, ab181602, Abcam, UK). Secondary horseradish peroxidase (HRP)-conjugated antibodies were used at a dilution of 1:5000: goat anti-rabbit HRP (12–348, Millipore, USA) and goat anti-mouse HRP (sc-2005, Santa Cruz Biotechnology, USA). Blots were visualized using enhanced chemiluminescence detection reagents with a Chemidoc™ Touch Imaging System (Bio-Rad, Hercules, CA, USA).

**References**

1. Ellenbroek B A Cools A R. Early maternal deprivation and prepulse inhibition: the role of the postdeprivation environment. *Pharmacol Biochem Behav.* 2002;73:177-184*.*

2. Au A, Feher A, McPhee L, et al. Estrogens, inflammation and cognition. *Front Neuroendocrinol.* 2016;40:87-100*.*

3. Galea L A M, Frick K M, Hampson E, Sohrabji F, Choleris E. Why estrogens matter for behavior and brain health. *Neurosci Biobehav Rev.* 2017;76:363-379*.*

4. Xu S, Hao K, Xiong Y, et al. Capsaicin alleviates neuronal apoptosis and schizophrenia-like behavioral abnormalities induced by early life stress. *Schizophrenia (Heidelb).* 2023;9:77*.*

5. Starowicz K, Nigam S, Di Marzo V. Biochemistry and pharmacology of endovanilloids. *Pharmacol Ther.* 2007;114:13-33*.*

6. Abbas M A. Modulation of TRPV1 channel function by natural products in the treatment of pain. *Chem Biol Interact.* 2020;330:109178*.*

7. Kong W L, Peng Y Y, Peng B W. Modulation of neuroinflammation: Role and therapeutic potential of TRPV1 in the neuro-immune axis. *Brain Behav Immun.* 2017;64:354-366*.*

8. Huang J, Huang H, Liu M, Yang W, Wang H. Involvement of the TRPV1 receptor and the endocannabinoid system in schizophrenia. *Brain Res Bull.* 2024;215:111007*.*

9. Kraeuter A K, Guest P C, Sarnyai Z. The Open Field Test for Measuring Locomotor Activity and Anxiety-Like Behavior. *Methods Mol Biol.* 2019;1916:99-103*.*

10. Hao K, Chen F, Xu S, et al. The role of SIRT3 in mediating the cognitive deficits and neuroinflammatory changes associated with a developmental animal model of schizophrenia. *Prog Neuropsychopharmacol Biol Psychiatry.* 2024;130:110914*.*

11. Hao K, Wang H, Zhang Y, et al. Nicotinamide reverses deficits in puberty-born neurons and cognitive function after maternal separation. *J Neuroinflammation.* 2022;19:232*.*

12. Cohen S J, Munchow A H, Rios L M, et al. The rodent hippocampus is essential for nonspatial object memory. *Curr Biol.* 2013;23:1685-1690*.*

13. Imayoshi I, Sakamoto M, Ohtsuka T, et al. Roles of continuous neurogenesis in the structural and functional integrity of the adult forebrain. *Nat Neurosci.* 2008;11:1153-1161*.*

14. Kraeuter A K, Guest P C, Sarnyai Z. The Y-Maze for Assessment of Spatial Working and Reference Memory in Mice. *Methods Mol Biol.* 2019;1916:105-111*.*

15. Valsamis B Schmid S. Habituation and prepulse inhibition of acoustic startle in rodents. *J Vis Exp.* 2011;e3446*.*

16. Zhang J, Rong P, Zhang L, et al. IL4-driven microglia modulate stress resilience through BDNF-dependent neurogenesis. *Sci Adv.* 2021;7*.*

17. Jiang X, Yi S, Liu Q, et al. Asperosaponin VI ameliorates the CMS-induced depressive-like behaviors by inducing a neuroprotective microglial phenotype in hippocampus via PPAR-gamma pathway. *J Neuroinflammation.* 2022;19:115*.*
